# Transcriptome Analysis Reveals the Genes Involved in Growth and Metabolism in Muscovy Ducks

**DOI:** 10.1155/2021/6648435

**Published:** 2021-04-17

**Authors:** Xingxin Wang, Yingping Xiao, Hua Yang, Lizhi Lu, Xiuting Liu, Wentao Lyu

**Affiliations:** ^1^College of Animal Science, ZheJiang A&F University, Hangzhou, China; ^2^State Key Laboratory for Managing Biotic and Chemical Threats to the Quality and Safety of Agro-Products, Institute of Agro-Product Safety and Nutrition, Zhejiang Academy of Agricultural Sciences, Hangzhou, China; ^3^Institute of Animal Husbandry and Veterinary Science, Zhejiang Academy of Agricultural Sciences, Hangzhou, China

## Abstract

Muscovy ducks are among the best meat ducks in the world. The objective of this study was to identify genes related to growth metabolism through transcriptome analysis of the ileal tissue of Muscovy ducks. Duck ileum samples with the highest (H group, *n* = 5) and lowest (L group, *n* = 5) body weight were selected from two hundred 70-day-old Muscovy ducks for transcriptome analysis by RNA sequencing. In the screening of differentially expressed genes (DEGs) between the H and L groups, a total of 602 DEGs with a fold change no less than 2 were identified, among which 285 were upregulated and 317 were downregulated. Gene Ontology (GO) enrichment analysis and Kyoto Encyclopedia of Genes and Genomes (KEGG) analysis revealed that glutathione metabolism, pyrimidine metabolism, and protein digestion and absorption processes played a vital role in regulating growth and metabolism. The results showed that 7 genes related to growth and metabolism, namely, *ANPEP*, *ENPEP*, *UPP1*, *SLC2A2*, *SLC6A19*, *NME4*, and *LOC106034733*, were significantly expressed in group H, which was consistent with the phenotype results. The validation of these 7 genes using real-time quantitative PCR results indicated that the expression level of *ENPEP* was significantly different between the H and L groups (*P* < 0.05). This study provides a theoretical basis for exploring the influence of the ileum on growth and metabolism in ducks.

## 1. Introduction

Duck meat is one of the most important meat sources in Asia with a high content of essential unsaturated fatty acids and is healthier than pork. It is popular in China and Southeast Asian countries. Native to the tropical regions of South and Central America, Muscovy ducks (*Cairina moschata*) have been gradually domesticated since their introduction into China. Because of their good meat quality, low adipose tissue content, high lean meat rate, and high protein content [1], Muscovy ducks have become an excellent meat duck in China.

At present, there are two ways to regulate the growth and metabolism of poultry. The first is to change the nutrient level in the diet. Gonzalez-Esquerra and Leeson [2] showed that high-protein diets increased daily body weight gain in broilers. Changing the structure of the intestinal flora is another way to modulate the growth and metabolism process. Many studies have shown that regulating the structure of the intestinal flora can indeed affect the growth of animals. Dietary supplementation with *Lactobacillus acidophilus* can change the structure of the intestinal microflora and thus increase body weight gain in Ross broiler chickens [3]. *Lactobacillus* fed to poultry reduces colonization by human foodborne pathogens such as *Campylobacter*, *Clostridium difficile*, and *Salmonella* in the gastrointestinal tract and promotes the growth of poultry [4]. The first two methods lead to changes in gene expression, which is the fundamental reason why they can regulate the growth and metabolism of poultry. Food intake, energy expenditure, and energy balance are regulated by cellular and molecular mechanisms. For example, a methionine deficient diet decreasing the body weight gain and increasing feed conversion ration might be due to the gene expression alteration of amino acid transporters in different tissues of chickens, such as Sodium-coupled Neutral Amino Acid Transporter (*SNAT1*), Aromatic Amino Acid Transporter (*TAT1*), and so on (Fagundes et al., 2020).

Transcriptome sequencing, also known as RNA sequencing (RNA-seq), is a technique used to explore and quantitatively describe the differences in gene types and expression levels at a global level [5]. It could also intuitively link changes at the gene level with phenotypic changes. With the development of this technique, livestock genetics and breeding have been greatly improved. The genetic mechanism affecting traits can be studied at the molecular level to select candidate genes, which will be helpful for breeding improvement in Muscovy ducks. Wang et al. used RNA-seq to analyze the caecum of chickens in an *E. tenella* infection group and an uninfected group and found that the *ANGPTL4*, *MAPK10*, and *CD44* genes played an important role in the invasion of *E. tenella* [6]. Chen et al. conducted transcriptome analysis on the breast muscles of fast- and slow-growing chickens and revealed that the *SNCG*, *PLPPR4*, and *VAMP1* genes were related to growth and metabolism in Jinghai yellow chickens [7].

The energy used for growth and metabolism in animals is mainly obtained from the digestion of nutrients in the small intestine. As the third portion of the small intestine, the ileum functions to absorb nutrients from foods and transfer them into the bloodstream of the body to provide energy and essential nutrients to the host. Consequently, genes expressed in the ileum are key to modulating the growth and metabolism of animals. To date, few studies have identified the key genes involved in regulating the growth and development of ducks. The intestine is essential for absorbing nutrients in the body. This study analyzed the ileal transcriptome of Muscovy ducks to identify the genes related to growth and metabolism. By doing so, it provides a theoretical basis for the genetic regulation of duck growth and metabolism.

## 2. Materials and Methods

### 2.1. Animals and Sampling

The animal processes in this study were approved by the Animal Care Committee of Zhejiang Academy of Agriculture Sciences (ZAASDLSY2019-1910). All the ducks were obtained from Hewang Poultry Industry Co., Ltd. (Lanxi, China). A total of 200 healthy Muscovy ducks, which were randomly selected from a population of 5000 70-day-old ducks, were weighed individually. Among these 200 ducks, the 5 ducks with the highest body weight and 5 ducks with the lowest body weight were set as the H group and the L group, respectively. After the ducks were slaughtered, the ileum segments were collected, immediately frozen in liquid nitrogen, and then stored at -80°C until RNA isolation.

### 2.2. RNA Extraction and cDNA Synthesis

Total RNA was isolated from the ileum segments, purified using a TRIzol® Plus RNA Purification Kit (Invitrogen, USA), and treated with a RNase-Free DNase Set (Qiagen, USA) according to the manufacturer's instructions. RNA purity was monitored by a NanoPhotometer® spectrophotometer (IMPLEN, USA) using OD260/280 and OD260/230 ratios. The quantity of RNA was determined by a NanoDrop spectrophotometer (Thermo Fisher Scientific Inc., USA). The integrity and contamination of RNA samples were checked by 1% agarose gel electrophoresis. The integrity of RNA samples was evaluated using the RNA Nano 6000 Assay Kit of the Agilent Bioanalyzer 2100 system (Agilent Technologies, USA).

### 2.3. Library Construction and Sequencing

The qualified RNA samples with the RIN (Supplementary Table 1) no less than 6 were used to construct cDNA sequencing libraries of the ileal transcriptome of Muscovy ducks using the NEBNext® Ultra™ RNA Library Prep Kit for Illumina® (NEB, USA) following the manufacturer's recommendations. After cluster generation by the cBot Cluster Generation System using a TruSeq PE Cluster Kit v3-cBot-HS (Illumina), the libraries were sequenced on an Illumina HiSeq 6000 platform for the generation of 150 bp paired-end reads.

### 2.4. Data Processing and Transcriptome Assembly

Raw reads were first processed through in-house Perl scripts to obtain clean reads by removing reads containing adapters, reads containing poly-N sequences, and low-quality reads. These reads were used for the *de novo* assembly using Trinity [8] software. The outputs of Trinity are sequences called unigenes that can form either clusters in which the similarity among sequences is >70% or unigenes that are unique and form the singletons [9]. The transcriptional sequences obtained by Trinity splicing were used as the reference genome (Ref) for subsequent analysis. Clean reads of each sample were mapped on the Ref to filter out the reads with a comparison quality value lower than 10, the reads on the unpaired comparison, and the reads in multiple regions of the genome in order to get mapping reads for each sample. The raw transcriptome read data are available in the SRA database under accession number PRJNA669499.

### 2.5. Differential Gene Expression Analysis

Differential expression analysis of the H and L groups was performed using the DESeq R package (1.10.1). DESeq provides statistical routines for determining differential expression in digital gene expression data using a model based on the negative binomial distribution. The resulting *P* values were adjusted using Benjamini and Hochberg's approach for controlling the false discovery rate. Genes with an adjusted *P* value < 0.05 found by DESeq were assigned as differentially expressed.

### 2.6. GO and KEGG Enrichment Analysis of Differentially Expressed Genes

Gene Ontology (referred to as GO, http://www.geneontology.org/) enrichment analysis of the differentially expressed genes (DEGs) was implemented by the GOseq R package-based Wallenius noncentral hypergeometric distribution [10], which can adjust for gene length bias in DEGs.

The Kyoto Encyclopedia of Genes and Genomes (referred to as KEGG, http://www.genome.jp/kegg/) [11] is a database resource for understanding the high-level functions and utilities of biological systems, such as cells, organisms, and ecosystems, from molecular-level information, especially large-scale molecular datasets generated by genome sequencing and other high-throughput experimental technologies. We used KOBAS [12] software to test the statistical enrichment of DEGs in KEGG pathways.

### 2.7. Real-Time Quantitative PCR (RT-qPCR) Analysis

To demonstrate the repeatability and precision of the RNA-seq gene expression data derived from the Muscovy duck ileum libraries, a CFX384 multiple real-time fluorescence quantitative PCR instrument was used for RT-qPCR analysis. The first-strand cDNA was synthesized from 300 ng RNA for each sample using iscript cDNA synthesis kit (Bio-Rad) in a total volume of 4 *μ*L. After ten times dilution of cDNA, the RT-qPCR system (20 *μ*L) was as follows: Power SYBR® Green Master Mix, 10 *μ*L; gene-specific upstream and downstream primers (10 *μ*mol/L), 0.5 *μ*L; sterile water, 8 *μ*L; and cDNA template, 1 *μ*L. The reaction conditions were as follows: 95°C for 1 min, followed by 40 cycles of 95°C for 15 sec and 63°C for 25 sec (for collecting fluorescence data). The reaction for each sample was repeated three times, and the relative expression level of each gene was determined using the 2^-*ΔΔ*Ct^ method with *GAPDH* as the internal reference gene (Livak and Schmittgen, 2001). The primers used for quantification in the study were designed using Primer-BLAST on the NCBI website (https://www.ncbi.nlm.nih.gov/tools/primer-blast/). The gene information for real-time PCR is shown in Table 1.

### 2.8. Statistical Analysis

Data are expressed as the mean ± standard error of mean (SEM). All statistical analyses were performed in SPSS version 23. The difference between the two groups was analyzed by unpaired two-tailed Student's *t*-test and considered significant when the *P* value was no more than 0.05.

## 3. Results

### 3.1. The Phenotype of Muscovy Ducks

**Figure 1:**
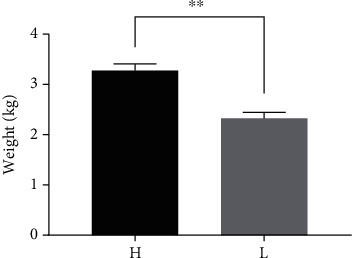
The average body weight of ducks in the H and L groups (*n* = 5). “∗∗” indicates significant differences (*P* < 0.01).

The body weight of the H group was 3.290 ± 0.138 kg, and that of the L group was 2.323 ± 0.117 kg. The body weight at the beginning and the end of the experiment and average daily gain is shown in Supplementary Table 2. The average body weight of the H group was approximately 1.4 times than that of the L group, showing a significant difference (*P* < 0.01) (Figure 1).

### 3.2. Overview of the Muscovy Duck Ileum Transcriptome Data

To detect differences in mRNA expression between the ducks with high and low body weight, we generated RNA-seq data from the ducks in the H and L groups. The average number of original reads for 10 samples was 52,472,860 (NCBI accession number PRJNA669499) After quality control of the original reads, there was an average of 51,479,286 clean reads for each sample, accounting for 98.11% of the original reads. The Q30 value is the percentage of bases for which the recognition accuracy exceeds 99.9%. The average Q30 value in the present study was 91.30%. The average data size was over 6.0 G, and only one sample was lower than 6G but still close. The percentage of reads aligned to the unique location of the reference genome was 79.27% to 81.08% among the clean reads, and the average mapping rate of clean reads mapped to reference genes was 80.11% (Table 2).To assess intergroup differences and intragroup sample duplication, we performed PCA analysis on FPKM and read count of all samples, and the results showed intragroup aggregation and intergroup isolation (Supplementary Figure 1). In summary, the sequencing data qualified for subsequent data analysis.

### 3.3. DEG Analysis

**Figure 2:**
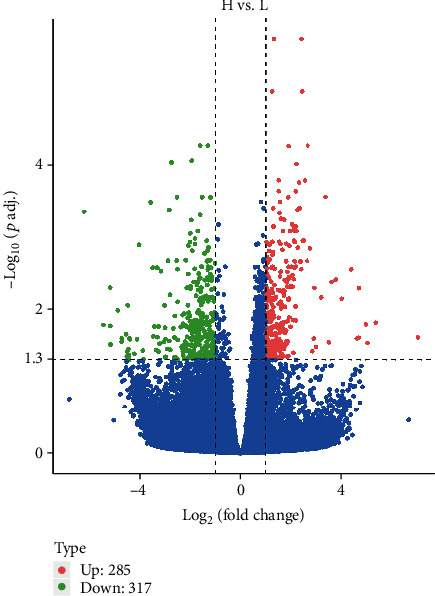
Volcano plot of the total expression of genes in both the H and L groups. The *x*-axis represents the log_2_(fold change) values for gene expression, and the *y*-axis represents the -log_10_(*P* value) (padj in the figure represents the *P* value; *P* < 0.5 indicates −log_10_(padj) > 1.3). Each dot represents a gene identified from the RNA-seq data. Red dots indicate 285 upregulated DEGs, green dots indicate 317 downregulated DEGs, and blue dots indicate 30,915 nondifferentially expressed genes.

A total of 31,517 genes were annotated in 7 databases, namely, Nr, Nt, Pfam, KOG/COG, Swiss-prot, KEGG, and GO. With *P* < 0.05 and ∣log‐2fold change | >1 as the threshold, a total of 602 DEGs were identified including 285 upregulated genes and 317 downregulated genes (Figure 2). A hierarchical cluster analysis was performed for the DEGs. We calculated the distance between the samples using the expression of different genes in each sample and determined the correlation between the samples.

**Figure 3:**
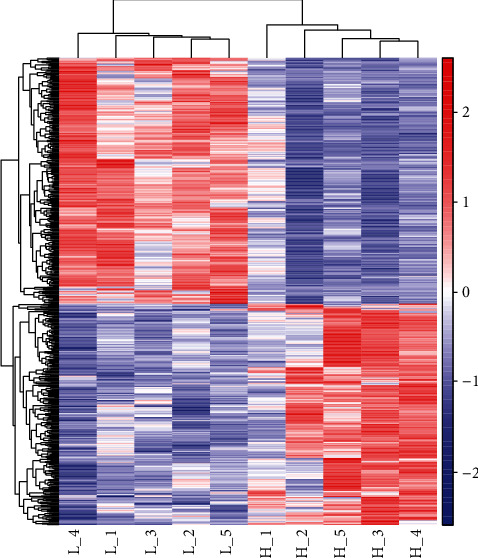
Ileal tissue expression profiles of 602 differentially expressed genes (DEGs) in the H vs. L group. The colour scale represents Fragments Per Kilobase of exon model per Million mapped fragment- (FPKM-) normalized log_10_(transformed counts). The horizontal bars represent genes. Each vertical column represents a sample, and sample names are as indicated. Red indicates upregulated genes, while blue indicates downregulated genes.

To identify DEGs in the ileums of H and L ducks, cluster analysis was performed. As shown in Figure 3, the high-weight and low-weight individuals were all clustered together, which illustrated the accuracy and reliability of the samples (Figure 3).

### 3.4. Functional Enrichment Analysis of DEGs

**Figure 4:**
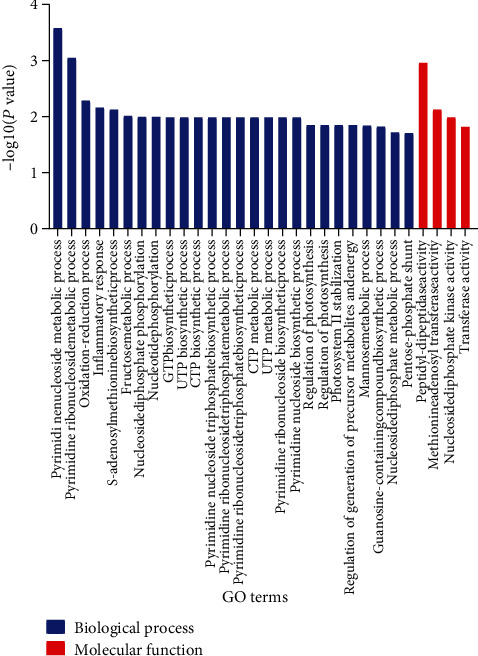
GO term enrichment analysis of differentially expressed genes (DEGs), with the 30 top terms obtained by GO enrichment. Gene classification was based on Gene Ontology for DEGs in the ileums of high-body weight (H) and low-body weight (L) ducks. The *x*-axis represents GO terms, and the *y*-axis represents the cluster frequency of the DEGs. The top 30 GO terms included 26 terms for biological process and 4 terms for molecular function.

To further elucidate the functional roles of the 602 DEGs, GO and KEGG pathway enrichment analyses were performed to search for significantly overrepresented categories. GO terms belonged to three categories, namely, biological process, cellular component, and molecular function, and 114 terms (*P* < 0.05) were significantly enriched in the three categories. The top 30 terms (including 26 terms for biological process and 4 terms for molecular function) were further analyzed to determine the associated regulatory functions (Figure 4). Within the biological process, oxidation-reduction process (GO: 0055114) was a considerably broad GO term, with more than 6% of the candidate genes being annotated within the term. The *NME4* and *LOC106034733* genes also participated in growth and development metabolism and were associated with 17 GO terms (including 16 terms for biological process and 1 term for molecular function). In addition, these 2 genes were also enriched in the KEGG pathway analysis (Table 3).

**Figure 5:**
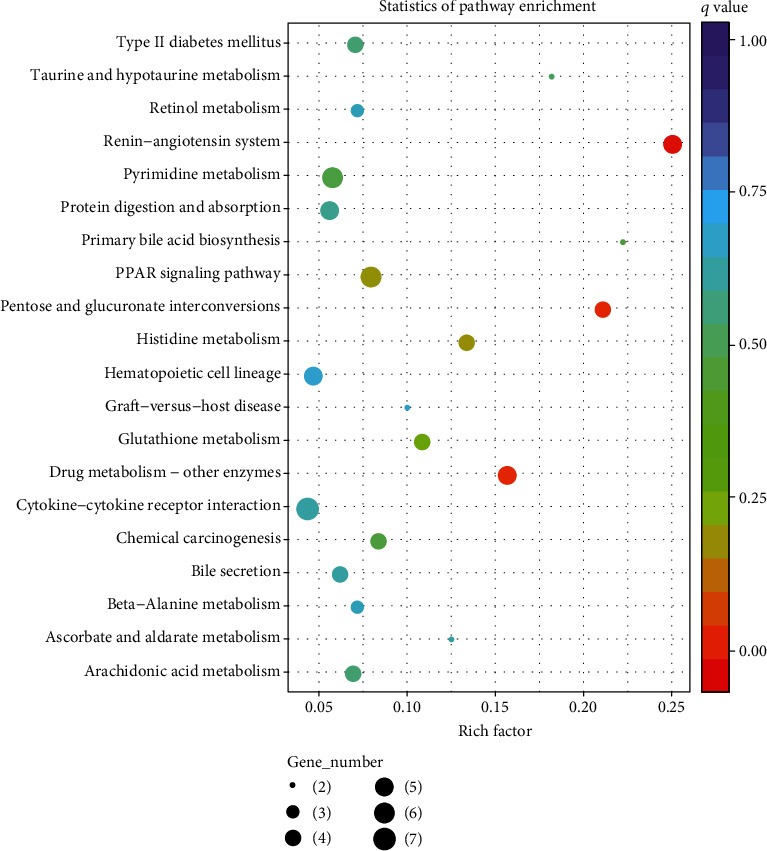
KEGG pathway enrichment analysis of differentially expressed genes (DEGs) with the top 20 pathways obtained by KEGG enrichment. The size of the dots indicates the number of expressed genes in the pathways, and the colour of the dots represents the *q* value of the pathway.

The 602 DEGs were also integrated into the KEGG pathway database, and a total of 17 pathways (*P* < 0.05) were significantly enriched (Figure 5). Among them, glutathione metabolism (ko00480), pyrimidine metabolism (ko00240), and protein digestion and absorption (ko04974) were related to growth metabolism. Moreover, the genes *ANPEP*, *ENPEP*, *UPP1*, *SLC2A2*, and *SLC6A19*, which are responsible for growth metabolism, were highly enriched in the KEGG pathway analysis (Table 3).

### 3.5. RT-qPCR Validation

**Figure 6:**
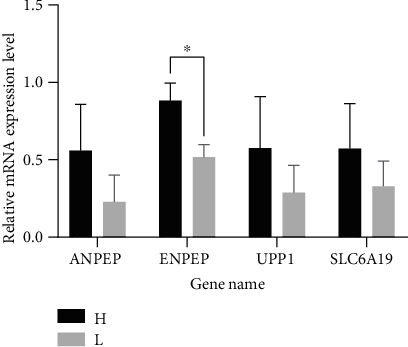
Validation of 4 DEGs between the H and L ducks by RT-qPCR. The *x*-axis denotes the DEGs name, and the *y*-axis denotes the log2 fold change derived from RT-qPCR.

To verify the RNA-seq expression results, we further determined the expression level of 7 growth-related genes in the ileum of ducks from H and L groups (*n* = 5), namely, *ANPEP*, *ENPEP*, *UPP1*, *SLC2A2*, *SLC6A19*, *NME4*, and *LOC106034733*, using RT-qPCR analysis. These 7 genes related to growth metabolism were selected from the KEGG pathways and the top 30 GO terms that were significantly enriched in relation to fat metabolism and growth metabolism. Interestingly, these DEGs were all upregulated. Among these 7 genes, *SLC2A2*, *NME4*, and *LOC106034733* exhibited expression levels that were too low to be examined (Ct > 35). Although the expression levels of these 4 determined genes in the ileum were higher in the H ducks than in the L ducks, *ENPEP* was the only gene that showed a significant (*P* < 0.05) difference between the H and L groups (Figure 6).

## 4. Discussion

For meat ducks, growth traits are an important indicator and have always attracted much attention for the development of the livestock industry. The gut microbiota [13] and dietary energy level are the two most direct and commonly used parameters for the regulation of the growth and metabolism of ducks. Many studies have shown that different dietary energy levels have different effects on the growth and metabolism of animals. Zhang et al. (Zhang et al., 2012) found that the dietary energy level has an impact on the expression of hormone-sensitive lipase (*HSL*) in sheep, which might result in the alteration of sheep growth performance. Cho et al. (Cho et al., 2017) found that the expression of genes related to cell growth was affected by metabolizable energy levels in ducks. And there are studies that have found that dietary phosphate restriction up-regulates ileal fibroblast growth factor 15 (*Fgf15*) gene expression through 1,25-dihydroxyvitamin D (1,25(OH)_2_D) and vitamin D receptor (VDR) and might affect hepatic bile acid homeostasis (Nakahashi et al., 2014). Therefore, we could regulate the expression of related genes by adding feed additives or changing dietary energy level, so as to regulate the growth and metabolism of Muscovy ducks.

At present, some genes have been found to be involved with the growth performance in poultry. Insulin-like growth factors (*IGFs*) mediate the growth-promoting effect of growth hormone (*GH*). Insulin-like growth factor-I (*IGF-I*) is one of the members of the insulin-like growth factor family and one of the regulatory signals of cell growth and proliferation. It plays an important role in regulating the lean meat content during the growth of livestock [14]. Tang et al. found a positive correlation between *IGF-1* gene expression and muscle growth in geese [15]. The *IGF-1* gene is closely related not only to the body weight but also to the body size of poultry. Wang et al. showed that the *IGF-1* gene was the main gene controlling chicken body size traits [16]. *GHRL* and *GHSR* genes are important candidate genes for the growth and development of the body. Here, we also found several candidate genes related to growth metabolism in Muscovy ducks.

Aminopeptidases constitute a large family in the enzyme system, mainly playing a role in the final stage of peptide decomposition, because the peptide chains formed by the hydrolysis of various endonuclease enzymes or proteasomes become the smallest constituent units of proteins, that is, amino acids, after decomposition by aminopeptidase to achieve a balance between protein transformation and metabolism. Therefore, aminopeptidase plays an important role in the maturation, growth, and defence of biological cells and maintains the dynamic balance inside the cells [17]. First, the aminopeptidase-mediated cycling of foreign proteins itself provides effective nutrients for the body: high aminopeptidase activity was found in the animal digestive system, suggesting that aminopeptidase is involved in the degradation of the foreign proteins in food for bodily metabolism and some other life activities involving amino acids, which has an important significance for animal life activities. Both alanyl aminopeptidase, membrane (*ANPEP*) and glutamyl aminopeptidase (*ENPEP*) belong to the aminopeptidase family and are upregulated in the H group.

Uridine phosphorylase and its derivatives play an important role in biological nucleotide metabolism, cell signalling, and energy metabolism pathways. Therefore, uridine phosphorylase directly affects the proliferation and life activities of organisms. Uridine phosphorylase (*UPP1*) was upregulated in group H, but there are few studies on the role of UPP1 in regulating the growth and development of Muscovy ducks.

The solute carrier (*SLC*) superfamily mediates the transmembrane transport of various solutes between cells and the external environment or the transport within cells. The research results show that the *SLC* superfamily mainly has the following types of biological functions: first, it controls the uptake and transport of cellular nutrients or energy substances; second, it participates in the absorption of essential ions or micronutrients in the body; and third, it participates in transmembrane transport and transduction of neurotransmitter signals. *SLC* members involved in cell nutrition and energy substance intake account for the largest proportion in the entire superfamily and play an indispensable role in the growth and proliferation of cells and the normal operation of various basic metabolic activities. Solute carrier family 2 member 2 (*SLC2A2*) and solute carrier family 6 member 19 (*SLC6A19*) were both upregulated in the H group, where *SLC2A2* transports glucose to various tissues in the body and maintains blood sugar balance [18], and SLC6A19 is involved in central nervous system signalling [19].

*NME/NM23* nucleoside diphosphate kinase 4 (*NME4*) and nucleoside diphosphate kinase-like (*LOC106034733*) were upregulated in the H group. The protein product of both genes is nucleoside diphosphate kinase (*NDPK*). Recent studies have found that *NDPK* can regulate cell proliferation, differentiation, development, and apoptosis [20].

In recent years, intestinal microorganisms have gradually become a hot research topic, and identification of the role of the intestinal flora structure in the body and the mechanism of action has become a research hotspot. A large amount of research data has shown that regulating the gut microbial composition can lead to body weight change. By adding growth promoters to the diet, the structure of the intestinal flora of animals can be changed to regulate growth metabolism. These two methods ultimately regulate growth metabolism by regulating the expression of genes related to growth metabolism. Goto et al. [21] believed that growth traits are important quantitative traits that are affected by genes and the environment. The variation in growth traits in the H and L groups might be driven by the gut microbiota. The relationship between growth performance and the gut microbiota needs to be investigated in the future.

## 5. Conclusions

In summary, a total of 7 genes related to growth and metabolism, namely, *ANPEP*, *ENPEP*, *UPP1*, *SLC2A2*, *SLC6A19*, *NME4*, and *LOC106034733*, were screened in the ileal tissue. In addition, through transcriptome analysis of ileal tissue, glutathione metabolism, pyrimidine metabolism, and protein digestion and absorption processes were found to play a vital role. This study fills in the gaps in knowledge regarding the genes that regulate growth and metabolism in ileal tissue and provides a basis for further research on genes that affect the growth and regulation of Muscovy ducks.

## Figures and Tables

**Table 1:** Primers for RT-qPCR.

Gene name	Product size (bp)	TM (°C)	Amplification efficiency	Primer sequences (5′ to 3′)
*GAPDH*	141	60	97%	F: GGAGCTGCCCAGAACATTATC
				R: GCAGGTCAGGTCCACGACA
*ANPEP*	79	60	96%	F: CAGCACAGCCTGCTCCTA
				R: GTCGGTGTTCTGCCAGTGT
*ENPEP*	78	60	96%	F: GGGACAGGTGCTATGGAGAA
				R: GGCAGACTCGTTGGGATCAT
*UPP1*	96	60	98%	F: GGAGCCAAGATGGTGACTATC
				R: CCATACCGTGACTGACTGACAA
*SLC6A19*	99	60	97%	F: CCAAGATGCCTGTCTCACCTT R: CAAGCACACCTTCCATGTTTCC


**Table 2:** Summary of sequence quality from ileal transcriptome analysis of Muscovy ducks.

Sample	Read numbers	Clean reads	Clean bases	Clean ratio (%)	Q30 (%)	Mapped ratio (%)
L1	60034660	58918534	8.84G	98.14	91.7	79.27
L2	52432870	51437864	7.72G	98.10	90.9	79.66
L3	60126402	59023270	8.85G	98.17	91.49	80.36
L4	56190064	55189756	8.28G	98.22	91.66	79.75
L5	48307096	47524666	7.13G	98.38	91.54	79.99
H1	57930042	56778664	8.52G	98.01	90.93	79.49
H2	48433750	47687076	7.15G	98.46	90.37	81.08
H3	44137468	43411310	6.51G	98.35	91.82	80.95
H4	56480274	54980640	8.25G	97.34	90.92	79.98
H5	40655974	39841080	5.98G	98.00	91.63	80.61

**Table 3:** Information on seven differentially expressed genes associated with growth metabolism.

Gene names	NCBI ID	log2 fold change	*P* value	Description
*ANPEP*	110403644	1.71	1.72*E* − 05	Alanyl aminopeptidase, membrane
*ENPEP*	428771	1.69	4.25*E* − 07	Glutamyl aminopeptidase
*UPP1*	101791376	1.56	5.65*E* − 06	Uridine phosphorylase 1
*SLC2A2*	102090795	2.46	6.60*E* − 07	Solute carrier family 2 member 2
*SLC6A19*	106492854	1.96	1.05*E* − 05	Solute carrier family 6 member 19
*`NME4*	104831436	3.63	8.28*E* − 06	NME/NM23 nucleoside diphosphate kinase 4
*LOC106034733*	106034733	1.06	5.99*E* − 06	Nucleoside diphosphate kinase-like

Note: *P* value was calculated on read count for each gene between the H group and L group by unpaired two-tailed Student's *t*-test.

## Data Availability

The raw transcriptome read data are available in the SRA database under accession number PRJNA669499.

## Abbreviations

ACBP: Acyl-CoA-binding protein
ACOX2: Acyl-CoA oxidase 2
ANGPTL4: Angiopoietin-like 4
ANPEP: Alanyl aminopeptidase, membrane
AvBD9: Avian *β*-defensin 9
CD44: Cluster of differentiation 44
ENPEP: Glutamyl aminopeptidase
FABP2: Fatty acid-binding protein 2
FABP4: Fatty acid-binding protein, adipocyte
FABP5: Fatty acid-binding protein 5
GAPDH: Glyceraldehyde-3-phosphate dehydrogenase
GDF-8: Growth differentiation factor 8
GHSR: Growth hormone secretagogue receptor or ghrelin receptor
GO: Gene Ontology
IGF-I: Insulin-like growth factor-I
KEGG: The Kyoto Encyclopedia of Genes and Genomes
KOG/COG: Cluster of euKaryotic Orthologous Groups/Cluster of Orthologous Groups
LOC106034733: Nucleoside diphosphate kinase-like
MAPK10: Mitogen-activated protein kinases 10
NDPK: Nucleoside diphosphate kinase
NME4: NME/NM23 nucleoside diphosphate kinase 4
NPY: Neuropeptide Y
Nr: RefSeq nonredundant proteins
Nt: Nucleotide Sequence Database
Pfam: Protein sequence families
SLC2A2: Solute carrier family 2 member 2
SLC6A19: Solute carrier family 6 member 19
SLC7A9: Solute carrier family 7 member 9
UPP1: Uridine phosphorylase 1.

## Supplementary Materials

Supplementary 1 Supplementary Table 1. RNA integrity of each sample.

Supplementary 2 Supplementary Table 2. Muscovy duck phenotypes in the H and L groups.

Supplementary 3 Figure S1. Differences between transcriptome replicates of H and L group ducks based on principal component analysis. Note: the abscissa is the first principal component and the ordinate is the second principal component.
