# Supplementary material for: Transcriptome Analysis Reveals the Genes Involved in Growth and Metabolism in Muscovy Ducks
Source: Biomed Res Int. 2021 Apr 17;2021:6648435. doi: 10.1155/2021/6648435 (PMC8077732; doi:10.1155/2021/6648435)
Supplement: Supplementary 1 — Supplementary Table 1. RNA integrity of each sample. [file 6648435.f1.docx]

**Supplementary Table 1. RNA integrity of each sample**

| **Sample** | **RIN** |
| --- | --- |
| H1 | 6.8 |
| H2 | 6.4 |
| H3 | 6.6 |
| H4 | 6.6 |
| H5 | 7.0 |
| L1 | 6.4 |
| L2 | 7.6 |
| L3 | 6.4 |
| L4 | 6.8 |
| L5 | 6.6 |
