# Supplementary material for: Transcriptome Analysis Reveals the Genes Involved in Growth and Metabolism in Muscovy Ducks
Source: Biomed Res Int. 2021 Apr 17;2021:6648435. doi: 10.1155/2021/6648435 (PMC8077732; doi:10.1155/2021/6648435)
Supplement: Supplementary 2 — Supplementary Table 2. Muscovy duck phenotypes in the H and L groups. [file 6648435.f2.docx]

Supplementary Table 2. Muscovy ducks phenotypes in H and L groups.

| Group | 1-Day Weight(g) | 70-Day Weight (kg) | Daily Gain(g) |
| --- | --- | --- | --- |
| H1 | 46.92 | 3.30 | 46.47 |
| H2 | 46.17 | 3.25 | 45.77 |
| H3 | 47.45 | 3.19 | 44.82 |
| H4 | 48.35 | 3.49 | 49.10 |
| H5 | 48.87 | 3.13 | 43.94 |
| L1 | 45.29 | 2.34 | 32.78 |
| L2 | 44.81 | 2.32 | 32.50 |
| L3 | 49.07 | 2.39 | 33.44 |
| L4 | 47.48 | 2.13 | 29.75 |
| L5 | 44.31 | 2.44 | 34.15 |
