# Supplementary material for: Transcriptome Analysis Reveals the Genes Involved in Growth and Metabolism in Muscovy Ducks
Source: Biomed Res Int. 2021 Apr 17;2021:6648435. doi: 10.1155/2021/6648435 (PMC8077732; doi:10.1155/2021/6648435)
Supplement: Supplementary 3 — Figure S1. Differences between transcriptome replicates of H and L group ducks based on principal component analysis. Note: the abscissa is the first principal component and the ordinate is the second principal component. [file 6648435.f3.pdf]

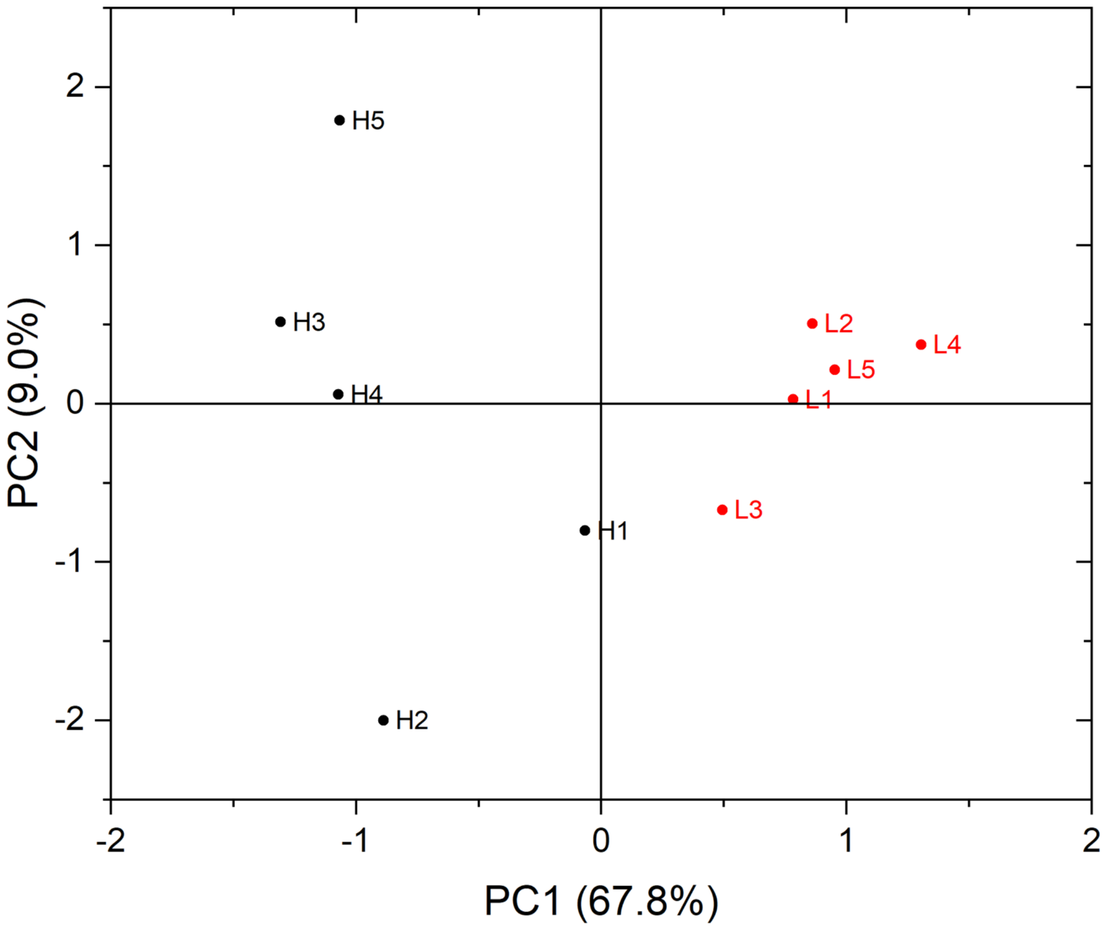

Figure S1. Differences between transcriptome replicates of H and L group ducks based on principal component analysis.

Note: the abscissa is the first principal component and the ordinate is the second principal component.
